# A high performance liquid chromatography tandem mass spectrometry protocol for detection of neurotransmitters in the rat brain tissue

**DOI:** 10.1016/j.mex.2023.102083

**Published:** 2023-02-18

**Authors:** Di Dai, Qian Qin, Xinyu Zhu, Qiuyuan Fang, Xianzong Meng, Lin Bai, Guang Yang, Ling Shan, Chunqing Liu

**Affiliations:** a Technology Centre of Dalian Customs District P.R. China; b College of Medical Laboratory, Dalian Medical University, Dalian, Liaoning 116044, China; c The First Affiliated Hospital of Dalian Medical University, China, Dalian, Liaoning 116011, China; d Dept. Neuropsychiatric Disorders, Netherlands Institute for Neuroscience, an Institute of the Royal Netherlands Academy of Arts and Sciences, Meibergdreef 47 1105BA Amsterdam, the Netherlands

**Keywords:** HPLC-MS/MS, HPLC-MS/MS, Neurotransmitter, Rat, Striatum, Hippocampus, Parkinson's disease, Alzheimer's disease

## Abstract

The detection of neurotransmitters has extensively been applied to the study of the pathogenesis, diagnosis, and therapeutic effect of drugs on many neuropsychiatric diseases. High-performance liquid chromatography-tandem mass spectrometry (HPLC-MS/MS) has been employed to determine neurotransmitters levels due to its distinct advantages. However, neurotransmitter detection still presents some challenges. A rapid and sensitive HPLC-MS/MS protocol has been established in our lab, which can simultaneously detect 5 neurotransmitters with an easy pretreatment procedure. The protocol provides demanded reference value for the lab using an Agilent HPLC-MS/MS system with a triple quadrupole analyzer.

## Graphical abstract

**Figure:**
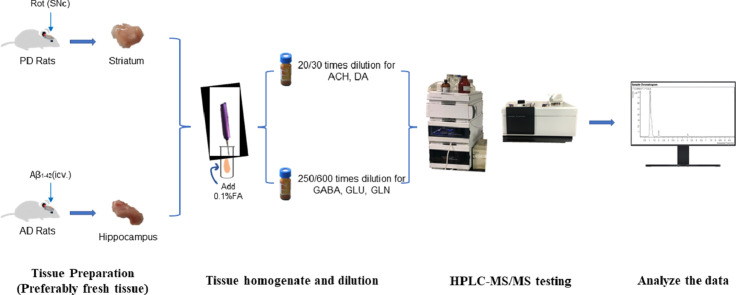

Specifications table

**Table:** 

Subject area:	*Neuroscience*
More specific subject area:	*Neurotransmitter detection*
Name of your protocol:	*A high performance liquid chromatography tandem mass spectrometry protocol for detection of neurotransmitters in the rat brain tissue*
Reagents/tools:	• *Dopamine hydrochloride (Cat.H8502, Sigma-Aldrich, St. Louis, USA)* • *ACH (Cat.B24126, Shanghai Yuanye Bio-Technology Co., Ltd, China)* • *GABA (Cat.A2129, Sigma-Aldrich, St. Louis, USA)* • *GLU (Cat.B21916, Shanghai Yuanye Bio-Technology Co., Ltd, China)* • *GLN (Cat.PHR1125, Sigma-Aldrich, St. Louis, USA)* • *Rotenone (Cat.R8875, Sigma-Aldrich, St. Louis, USA)* • *Bate-Amyloid (1–42) human (Cat.52487, Shanghai Gill Biochemical, China)* • *Formic acid (OCEANPAK Guangzhou, China)* • *Acetonitrile (OCEANPAK Guangzhou, China)* • *Agilent1290 HPLC system, coupled to an Agilent 6430 mass spectrometer (Agilent, Santa Clara, CA, USA)* • *Purospher RP-18 end capped (150 × 2* *mm i.d., 5* *µm) Hibar RT 150–2 (Merck KGaA, Darmstadt, Germany)*
Experimental design:	*In this paper, we established a rapid and sensitive HPLC-MS/MS protocol using an Agilent-1290 HPLC system coupled with an Agilent-6430 mass spectrometer to measure 5 neurotransmitters in rat brain tissue simultaneously. Subsequently, the protocol was applied to detect these neurotransmitters in brain tissues from rat models of Parkinson's disease (PD) and Alzheimer's disease (AD).*
Trial registration:	*NA*
Ethics:	*We followed the Guideline of Animal Experimentation of the Animal center of Dalian Medical University, PR, China. The protocol was approved by the Animal Ethics Committee of the University (Certification* No:211003700001575). *All efforts were made to minimize pain or discomfort to the animals.11 adult male Sprague-Dawley (SD) rats were included in the PD experiment and 10 female aged SD rats were used in AD experiment. Because there were two independent experiments, the effect of gender on the results were not discussed.*
Value of the Protocol:	• *Simultaneously detect 5 neurotransmitters* • *An easy pretreatment procedure* • *For the lab using an Agilent HPLC-MS/MS system with a triple quadrupole analyzer*

## Description of protocol

A large number of studies have demonstrated that multiple neurotransmitter systems are involved in many neurological disorders including Parkinson's disease (PD) and Alzheimer's disease (AD) [1], [2], [3]. The neuroactive amino acids play crucial functions in the brain [4]. Several neurotransmitters such as Acetylcholine (ACH), Dopamine (DA), Hydroxytryptamine (5-HT), γ-Aminobutyric acid (GABA) and Glutamic acid (Glu), have been shown to be significantly altered in PD and AD brains [5], [6], [7], suggesting their potential use as diagnostic markers for these two diseases [8]. In addition, these neurotransmitters play crucial functions in the brain, for example motor functions, cognition and emotion. Some of those neurotransmitters have been shown to also be altered in PD and AD. High performance liquid chromatography tandem mass spectrometry (HPLC-MS/MS) methods have been previously employed to measure neurotransmitters [9,10]. However, it always takes a lot of trial and error to establish a stable and reliable protocol. For an Agilent 1290 HPLC system coupled with an Agilent-6430 mass spectrometer, we, therefore, developed a rapid, stable and sensitive protocol with simple pre-treatment in our lab using HPLC-MS/MS to simultaneously measure 5 neurotransmitters including ACH, DA, GABA, Glu-and l-glutamine (GLN) in rat brain sample. Subsequently, we validated this protocol in rat PD and AD models, respectively.

### Chemicals and standards

The standardized chemicals of DA (Cat.H8502), GABA (Cat.A2129), GLU (Cat.B21916) and GLN (Cat.PHR1125) for calibrations were purchased from Sigma-Aldrich, St. Louis, USA. The ACH standardized chemicals (Cat.B24126) were purchased from Yuanye Biological Technology, Shanghai, China. The purity of these reference standards was more than 98%. ACH, DA, GABA, GLU and GLN were prepared by 0.1% formic acid (FA) for the stock (1 mg/mL) and the following working standard solutions. To avoid oxidation of DA, 0.2 mg/mL Vitamine C also dissolved in 0.1%FA.The HPLC-grade FA (Analytic grade, 88%) and acetonitrile (Analytic grade ≥ 99.9%) were purchased from OCEANPAK (Guangzhou, China). Rotenone (Cat.R8875) was purchased from Sigma-Aldrich, St. Louis, USA, and the Aβ 1–42 (Cat.52487) was purchased from Shanghai Gill Biochemical, China.

### Development of calibration curves

Stock solutions of standards were mixed and diluted to a concentration of 10 μg/mL and the standard solutions at the concentration range of 0–400 ng/ml (0,10,20,50,100,200,400 ng/mL) were achieved as working solutions. The R^2^ values of four calibration curves were 0.9925 for ACH, 0.9993 for DA, 0.9998 for GABA, 0.9999 for GLU and 0.9998 for GLN, respectively.

### Sample preparation

The animal experimental work was carried out following the Guideline of Animal Experimentation of the Animal Center of Dalian Medical University, PR, China. The protocol was approved by the Animal Ethics Committee of the University. All efforts were made to minimize pain or discomfort to the animals. The characteristics of animals and housing conditions of male (for PD) and female (for AD) Sprague-Dawley (SD rats) have been described in detail before [11,12].

The rat PD model was generated according to our recently published article by unilateral injection of rotenone (12 μg) into the right substantia nigra pars compacta (SNpc) [7,8]. Three weeks after the rotenone injection, the rats were anesthetized with 10% chloral hydrate and perfused through the left ventricle with saline (the perfusion stopped once the lavage fluid from the right auricle became clear, approximately 8 min) and brains were taken out immediately and stored in −80 °C.

A female aged rat AD model was made according to published articles with modifications [13]. A fixed cannula was implanted in the right lateral ventricle (RV) (*L* = 1.5 mm; AP = 1.0 mm; DV = 3.8 mm) for soluble Aβ oligomers administration (3.36 μg for 3 weeks). Preparation of soluble Aβ 1–42 oligomers as the described protocol in the article of Ding YF [14].

The striata from PD animal models and the hippocampus from AD animal models were dissected and homogenized with a tissue homogenizer (5 μl 0.1% FA/mg brain tissue) for 30 s. Samples were then centrifuged at 14,000 rpm at 4 °C for 10 min. The supernatant was collected and filtered through a 0.22 μm filter (Jinteng test equipment Co., LTD., Tianjin, China). The filtered supernatant was then diluted 20 times for ACH and DA measurements, 250 times for GABA, GLU and GLN measurements in the striata in PD, while 30 and 600 times in the hippocampus derived from the AD rat model with a 0.1% FA solution. 20 μl of the samples were injected into the HPLC-MS/MS system by an autosampler.

### Instruments

The HPLC-MS analysis was performed on an Agilent-1290 HPLC system, coupled to an Agilent-6430 mass spectrometer (Agilent, Santa Clara, CA, USA). Chromatographic separation was conducted on an analytical column (Purospher RP-18 end capped (150 × 2 mm i.d., 5 µm) Hibar RT 150–2 (Merck KGaA, Darmstadt, Germany)). The samples were placed in an Agilent well plate and a 20 μl aliquot was automatically injected into the mobile phase.

### HPLC-MS/MS conditions

Chromatographic separation was achieved by mobile phases (solvent A: 0.1% aqueous FA; solvent B: acetonitrile) at a flow rate of 0.3 ml/min. The total analysis was 10 min, and it was operated with a gradient program as follows: 0–1 min: 2%B; 1–6min:2%- 90%B;6–7 min,90%B; 7–10min: 2%B. The column temperature was maintained at 35 °C.

The MS/MS detection was performed using multiple reaction monitoring (MRM), in the mode of positive electrospray ionization (ESI+). The ion spray voltage was set at 2500 V and the temperature was maintained at 350 °C. Gas1 and Gas2 were set at 20 psi and 15 psi. The mass transitions of the protonated precursor/product ion pairs that were used to record the selected ion mass chromatograms of all the chemicals were optimized. The parameters of ions and fragmentations are according to previous studies [15] and our parameters are listed in Table 1. The integration peak area of the multiple reaction monitoring transitions of each analysis was estimated using Mass Hunter Workstation Quantitative Analysis software (Agilent, Santa Clara, CA, USA).

**Table 1:** Ions and fragmentations used in multiple-reaction monitoring mode for five analytes.

Compound Name	Precursor ion (Da)	Product ion-1(Da)	Product ion-2(Da)	Dwell time (ms)	Fragment energy-1 (V)	Fragment energy-2 (V)	Collision energy-1 (V)	Collision energy-2 (V)	Cell Accelerator Voltage (V)	Polarity
ACH	146.2	87.2	43.5	20	50	50	16	27	3	positive
DA	154.1	137.1	119	50	50	50	10	10	3	positive
GABA	104.1	87	69	20	50	50	8	10	3	positive
GLU	148.1	102.1	84.1	20	80	80	8	15	3	positive
GLN	147	130	84	26	60	60	14	18	3	positive

ACH: Acetylcholine chloride; DA: Dopamine hydrochloride; GABA: γ-aminobutyric acid; GLN: l-Glutamine; GLU: Glutamic acid.

### Method validation

**Fig. 1:**
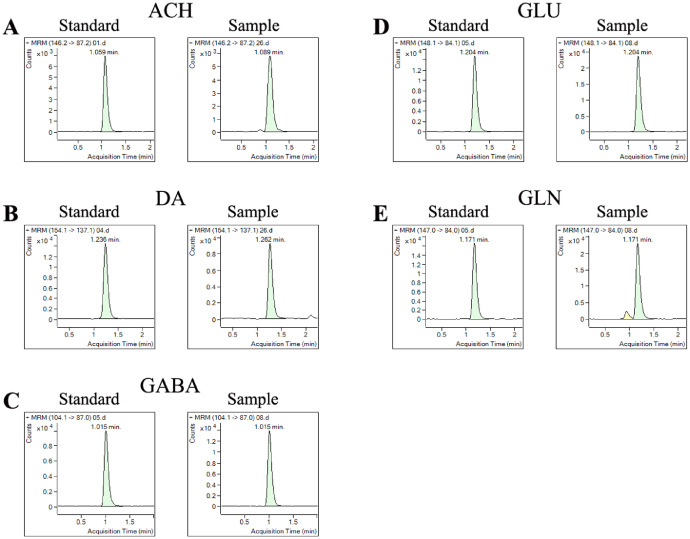
Typical multiple reaction monitoring chromatograms of 5 standards and neurotransmitters side by side. Acetylcholine (ACH)(A);Dopamine(DA)(B);γ-Aminobutyric acid (GABA); (C) Glutamic acid (Glu); (D) and l-glutamine (GLN)(E).

All standards and samples displayed good response values and representative chromatographic peaks are shown in Fig. 1. Because all of the analytes targets are endogenous metabolites, the determination of Limit of Detection (LOD), Limit of Quantitation (LOQ), Repeatability, the precision of intra-day and inter-day was carried out using standard mixtures. The recovery of the analyte was calculated as [A (mixture of sample and standard)-A(sample)]/A(standard) × 100%, in which A represents the relative peak area of analytes.

The results demonstrated that the LOD and LOQ of all five analytes were in the range of 0.26–3.77 and 0.88–12.58 ng/mL, which indicates that the present protocol is highly sensitive for ACH, GABA, GLU, and GLN, while the relative higher concentration (LOD 3.77 ng/ml, LOQ 12.58 ng/ml) in the sample is required for the detection of DA (Table 2). The variation (shown as Relative Standard Deviation (RSD)) of repeatability, intra-day precision and inter-day precision of all analytes should be lower than 15% [16] . In our experiments, all these parameters were lower than 5%. The optimal recovery should be between 80-120%. In our protocol, the recovery assay was determined at 3 (low to high) standard concentrations (5–100 ng/ml) (Table 3). The recovery of Ach, DA, GABA, GLU or GLN were almost falling well within the range.

**Table 2:** Summary of the limit of detection (LOD), the limit of quantification (LOQ), repeatability and the accuracy of intra-day/inter-day of five standards.

	LOD (ng/mL)	LOQ (ng/mL)	Repeatability (RSD%)	Intra-day (RSD%, *n* = 18, m/a/n)	Inter-day (RSD%, *n* = 18, D1-D3)
ACH	0.26	0.88	2.34	1.36	3.83
DA	3.77	12.58	2.37	0.38	3.33
GABA	1.47	4.90	2.27	0.64	2.74
GLU	0.88	2.94	2.64	1.56	3.30
GLN	0.84	2.80	2.96	1.33	4.62

ACH: Acetylcholine chloride; DA: Dopamine hydrochloride; GABA: γ-aminobutyric acid; GLU: Glutamic acid; GlN: l-Glutamine. The pass criteria of RSD: ≤15%.

**Table 3:** The recovery of five analytes with rat brain tissues.

Recovery% (Pass criteria:80–120%)			
ACH	113.91 (5 ng/ml)	110.99 (25 ng/ml)	108.32 (50 ng/ml)
DA	93.46 (25 ng/ml)	87.10 (50 ng/ml)	78.80 (100 ng/ml)
GABA	102.31 (5 ng/ml)	89.50 (50 ng/ml)	117.08 (100 ng/ml)
GLU	99.42 (5 ng/ml)	107.83 (25 ng/ml)	109.6 (50 ng/ml)
GLN	122.56 (5 ng/ml)	81.05 (25 ng/ml)	81.05 (50 ng/ml)

ACH: Acetylcholine chloride; DA: Dopamine hydrochloride; GABA: γ-aminobutyric acid; GLU: Glutamic acid; Gln: l-Glutamine.

### Applications to the animal model

To validate the efficacy of the present HPLC-MS/MS protocol, both PD and AD animal models were included in the validation. PD/AD rat models (*n* = 4–6) were made and their corresponding controls were included as well. The concentrations of 5 neurotransmitters in the striata of PD and the hippocampus of AD are shown in Tables 4 and 5. The studies of El-Naggar T and Fonseca BM provided similar concentrations with our data [17,18], which confirmed the reliability and reproducibility of our protocol.

**Table 4:** The levels of five analytes in the striatum of PD rat model induced by Rotenone.

			ACH (ng/mg)	DA (ng/mg)	GABA (ng/mg)	GLU (ng/mg)	GLN(ng/mg)
PD	Vehicle group	*n* = 5	5.66±4.25	3.16±0.85	438.53±165.92	426.57±31.07	610.19±190.23
	Rotenone group	*n* = 6	0.75±0.67⁎⁎	0.50±0.38⁎⁎	466.91±180.39	334.63±70.08*	433.39±60.56*

ACH: Acetylcholine chloride; DA: Dopamine hydrochloride; GABA: γ-aminobutyric acid; GLU: Glutamic acid; Gln: l-Glutamine.

⁎ p<0.05 compared with vehicle group.

⁎⁎ *p* < 0.01 compared with vehicle group. The data are shown as the mean ± S*.*E.M.

**Table 5:** The levels of four analytes in the hippocampus of AD rat model induced by Aβ1–42.

			A-CH (ng/mg)	DA (ng/mg)	GABA(ng/mg)	GLU(ng/mg)	GLN(ng/mg)
AD	Vehicle group	*n* = 4	2.17±0.16	1.17±0.02	1210.85±39.56	3586.46±169.7	1798.91±87.28
	Aβ group	*n* = 6	0.96±0.35*	1.19±0.02	1160.04±119.9	3886.55±294.66	1779.41±148.83

Ach: Acetylcholine chloride; DA: Dopamine hydrochloride; GABA: γ-aminobutyric acid; GLU: Glutamic acid; Gln: l-Glutamine.

⁎ p<0.05 compared with vehicle group. The data are shown as the mean ± S*.*E.M.

In PD rats, a decrease of a decrease of DA (15.82% of controls, *P* < 0.05), ACH (13.25% of controls, *P* < 0.05), GLU (78.45% of controls, *P* < 0.05) or GLN (71.03% of controls, *P* < 0.05) in the striatum was observed (Table 4). In AD rats, only ACH (44.24% of controls, *P* < 0.05) were decreased (Table 5). These results are consistent with previous literature in PD [19], [20], [21] and AD [22,23].

In conclusion, we established a rapid and sensitive HPLC-MS/MS protocol with simple pre-treatment to simultaneously measure ACH, DA and amino acid (GABA, GLU and GLN) neurotransmitters in rat brain tissue. Furthermore, the protocol was successfully validated in the striatal tissues of PD and hippocampal tissues of AD rat models.

## CRediT author statement

**Di Dai**: HPLC-MS/MS detection. **Qian Qin**: Supervision of HPLC-MS/MS detection. **Xinyu Zhu**: AD rat model. **Qiuyuan Fang**: PD rat model. **Xianzong Meng**: Development of calibration curves. **Lin Bai**: HPLC-MS/MS data analyses. **Guang Yang**: Samples preparation. **Ling Shan** and **Chunqing Liu**: Conceptualization,Writing-Reviewing and Editing.

## Funding

This work was supported by the 10.13039/501100005047Natural Science Foundation of Liaoning Province (2020-MS-254) awarded to Dr. CQ Liu. Stichting ParkinsonFonds (the Dutch Parkinson's foundation) and Friends of the Netherlands Institute for Neuroscience Foundation funded Dr. L Shan.

## Declaration of Competing Interest

The authors declare that they have no known competing financial interests or personal relationships that could have appeared to influence the work reported in this paper.

## Data Availability

Data will be made available on request.
